# Accelerated epigenetic age as a biomarker of cardiovascular sensitivity to traffic-related air pollution

**DOI:** 10.18632/aging.202341

**Published:** 2020-12-07

**Authors:** Cavin K. Ward-Caviness, Armistead G. Russell, Anne M. Weaver, Erik Slawsky, Radhika Dhingra, Lydia Coulter Kwee, Rong Jiang, Lucas M. Neas, David Diaz-Sanchez, Robert B. Devlin, Wayne E. Cascio, Kenneth Olden, Elizabeth R. Hauser, Svati H. Shah, William E. Kraus

**Affiliations:** 1 Center for Public Health and Environmental Assessment, US Environmental Protection Agency, Chapel Hill, NC 27709, USA; 2 Department of Civil and Environmental Engineering, Georgia Institute of Technology, Atlanta, GA 30332, USA; 3 Oak Ridge Associated Universities at the US Environmental Protection Agency, Chapel Hill, NC 27709, USA; 4 Department of Environmental Sciences and Engineering, Gillings School of Public Health, University of North Carolina, Chapel Hill, NC 27599, USA; 5 Institute for Environmental Health Solutions, University of North Carolina, Chapel Hill, NC 27599, USA; 6 Duke Molecular Physiology Institute, Duke University School of Medicine, Durham, NC 27710, USA; 7 Department of Psychiatry and Behavioral Sciences, Duke University School of Medicine, Durham, NC 27710, USA; 8 Department of Biostatistics and Bioinformatics, Duke University School of Medicine, Durham, NC 27710, USA; 9 Cooperative Studies Program Epidemiology Center, Durham Veterans Affairs Medical Center, Durham, NC 27705, USA; 10 Division of Cardiology, Department of Medicine, School of Medicine, Duke University, Durham, NC 27710, USA

**Keywords:** DNA methylation age, traffic, environmental sensitivity, cardiovascular disease, air pollution

## Abstract

Background: Accelerated epigenetic age has been proposed as a biomarker of increased aging, which may indicate disruptions in cellular and organ system homeostasis and thus contribute to sensitivity to environmental exposures.

Methods: Using 497 participants from the CATHGEN cohort, we evaluated whether accelerated epigenetic aging increases cardiovascular sensitivity to traffic-related air pollution (TRAP) exposure. We used residential proximity to major roadways and source apportioned air pollution models as measures of TRAP exposure, and chose peripheral arterial disease (PAD) and blood pressure as outcomes based on previous associations with TRAP. We used Horvath epigenetic age acceleration (AAD) and phenotypic age acceleration (PhenoAAD) as measures of age acceleration, and adjusted all models for chronological age, race, sex, smoking, and socioeconomic status.

Results: We observed significant interactions between TRAP and both AAD and PhenoAAD. Interactions indicated that increased epigenetic age acceleration elevated associations between proximity to roadways and PAD. Interactions were also observed between AAD and gasoline and diesel source apportioned PM_2.5_.

Conclusion: Epigenetic age acceleration may be a biomarker of sensitivity to air pollution, particularly for TRAP in urban cohorts. This presents a novel means by which to understand sensitivity to air pollution and provides a molecular measure of environmental sensitivity.

## INTRODUCTION

Air pollution continues to be a significant contributor to morbidity and mortality worldwide [1]. Concerningly, though air quality continues to improve worldwide, particularly decreases in particulate matter < 2.5 μm in diameter (PM_2.5_), a globally aging population may result in substantial segments of the population still experiencing significant environmental health risks as the elderly are highly sensitive to even low level of air pollution [2, 3]. This increased sensitivity is potentially due to breakdown in biological homeostasis at the cellular and organ system level accompanied by the accumulation of chronic disease and functional deficits. However, there is significant heterogeneity in how people age and accumulate the biochemical, functional, and clinical deficits which may contribute to increased sensitivity to environmental exposures.

Recently epigenetic (DNA methylation) age has emerged as a promising biomarker of "biological age" which, while correlated with chronological age, is more inherently tied to biological processes (e.g. alterations in DNA methylation) than chronological age itself [4]. DNA methylation-derived aging biomarkers have proven to be dynamic and responsive to the environment [5–7], and differences between DNA methylation age and chronological age, e.g. epigenetic age acceleration, are associated with mortality, cancer, obesity, and several other health outcomes [8–12]. Given its role as an indicator of biological age, DNA methylation-derived aging biomarkers may better function as indicators of heightened sensitivity to air pollution exposure than chronological age. To evaluate the role of DNA methylation age acceleration as an environmental sensitivity factor, we utilized the CATHGEN cohort, which had the necessary clinical outcomes, environmental exposures, and molecular data for analyses. CATHGEN participants were recruited from cardiac catheterization patients and have strong associations with air pollution, particularly traffic-related air pollutants [13–15].

## RESULTS

**Figure 1:**
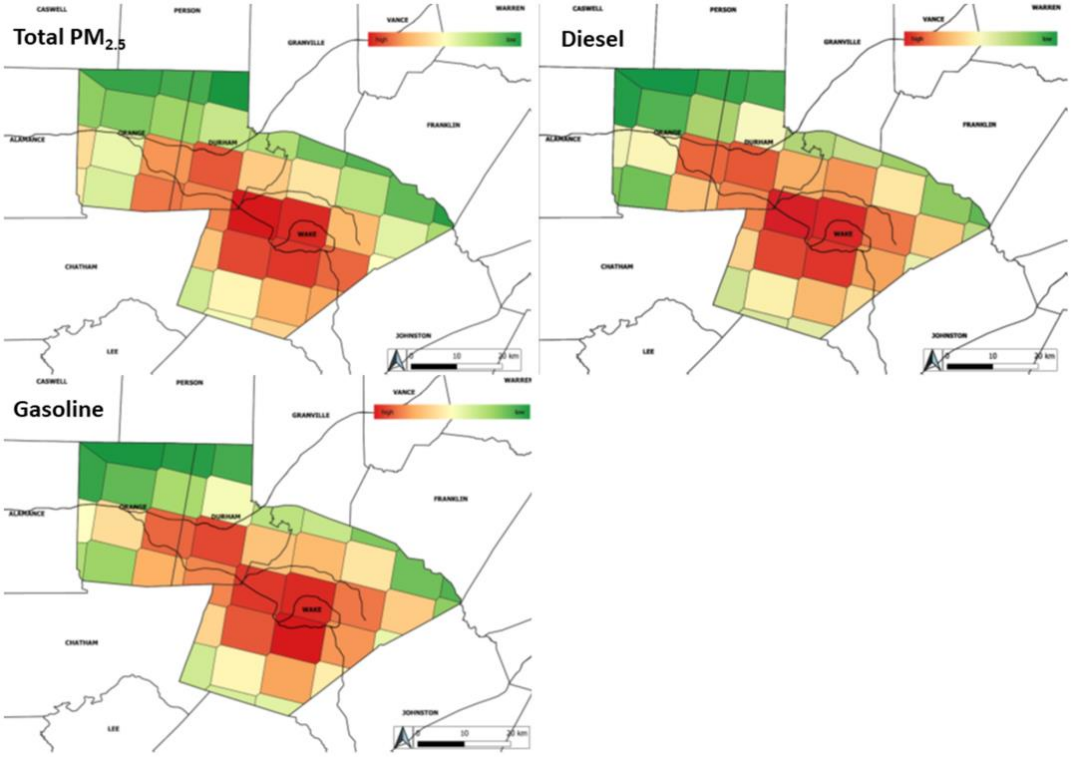
**Distribution of total PM_2.5_ and source-apportioned PM_2.5_ averaged over all study years.** gives the distribution of total PM_2.5_ mass as well as diesel and gasoline source-apportioned PM_2.5_. As expected for urban counties, the distribution of diesel and gasoline source-apportioned PM_2.5_ closely matches the distribution of total PM_2.5_. Lower levels of pollutants are given in green with high levels shading towards red.

Of the 563 total participants available for this analysis, 542 had the street-level geocoding necessary to estimate residential proximity to roadway, and 497 participants overlapped with the modeling time period for source apportioned PM_2.5_ (Table 1). Participants had an average age of 60.1 y and resided an average of 1.02 km from the nearest roadway. The average systolic blood pressure (SBP) was 146 mmHg while the average diastolic blood pressure (DBP) was 82.2 mmHg, slightly elevated readings which are likely driven by the 30.2% (170) participants with a history of hypertension. A total of 34 participants (6.0%) reported a history of peripheral arterial disease (PAD). The mean age acceleration difference (AAD) and phenotypic age acceleration difference (PhenoAAD) were 4.77 y, and -8.83 y respectively. Supplementary Figure 1 gives the distribution of AAD by PhenoAAD tertiles. Figure 1 gives the distribution of total PM_2.5_ as well as source apportioned PM_2.5_ for the sources examined in this analysis. We used neighborhood socioeconomic clusters to adjust for socioeconomic status in the analyses (Supplementary Table 1**)** **[**16**]**.

**Table 1:** Cohort description.

	**Mean**	**SD**	**N non-missing**	**IQR**
Age (y)	60.1	12.4	563	19.0
Body Mass Index (kg/m^2^)	30.6	7.53	556	8.26
AAD (y)	4.77	6.62	563	8.41
PhenoAAD (y)	-8.83	7.74	563	10.0
Distance to major roadway (km)	1.02	1.07	542	1.09
Total PM_2.5_ (μg/m^3^)	10.9	0.76	497	0.91
Diesel PM_2.5_ (μg/m^3^)	0.54	0.31	497	0.51
Gasoline PM_2.5_ (μg/m^3^)	0.55	0.16	497	0.26
Secondary Organic Carbon PM_2.5_ (μg/m^3^)	1.26	0.25	497	0.34
Biomass PM_2.5_ (μg/m^3^)	1.99	0.22	497	0.32
Diastolic Blood Pressure (mmHg)	82.2	14.0	539	18.0
Systolic Blood Pressure (mmHg)	146	24.8	539	36.0
	N	%	N non-missing	
Males	234	41.6	563	
Females	329	58.4	563	
African Americans	214	38.0	563	
European Americans	349	62.0	563	
Smokers	249	44.2	563	
Peripheral Arterial Disease	34	6.04	563	
Diabetes	162	28.8	563	
Hyperlipidemia	324	57.5	563	

Description of the study cohort. AAD = Age Acceleration Difference; mmHg = millimeters of mercury; IQR = interquartile range; PhenoAAD = Phenotypic Age Acceleration Difference; SD = standard deviation.

Our primary outcomes were PAD, SBP, and DBP. We report only the estimates from the more parsimonious full model as the effect estimates from both the full and clinical models were concordant (Supplementary Figure 2). Estimates for the clinical model can be found in Supplementary Table 2. As air quality and clinical practices can change from year to year, there can be potential confounding by year of assessment on the relationship between air quality and health outcomes. Our primary exposure, residential proximity to roadways, would be unaffected by this as it was assessed at a single point in time. The source apportioned PM_2.5_ did not have strong correlations with year (Supplementary Figure 3), an including year as a covariate did not modify associations (Supplementary Table 3). Thus, we kept the models as described in the methods.

We observed interactions between residential proximity to major roads and AAD for PAD, SBP, and DBP. For PhenoAAD we only observed interactions for DBP (Table 2). These associations did not differ when additionally adjusting for cell type proportions **(**Supplementary Table 4). When examining chronological age, we did not observe any interactions between chronological age and residential proximity to major roadways either with or without adjustment for AAD and PhenoAAD (Supplementary Table 5), highlighting that interactions with AAD and PhenoAAD are primarily driven by accelerated DNA methylation age as opposed to simply increased chronological age.

**Table 2:** Interactions between accelerated aging and residential proximity to roadways.

**Outcome**	**Accelerated aging measure**	**β**	**SE**	**P**
PAD	AAD	0.06	0.03	0.04
DBP	AAD	0.25	0.11	0.03
SBP	AAD	0.46	0.20	0.02
PAD	PhenoAAD	0.04	0.03	0.14
DBP	PhenoAAD	0.19	0.09	0.04
SBP	PhenoAAD	0.12	0.16	0.46

Interactions between the inverse-log transform of distance to major roadways (primary exposure) and each accelerated aging measure and outcome for the Full model (age, race, sex, sociodemographic cluster, and smoking adjusted). Estimates shown are all from the multiplicative interaction term between aging measure and inverse-log transform of distance to major roadways. For the binary outcome of PAD the regression coefficient represents the log-odds ratio for the interaction term. AAD = age acceleration difference; β = regression coefficient; DBP = diastolic blood pressure; PAD = peripheral arterial disease; SBP = systolic blood pressure; SE = standard error.

We additionally examined associations for traffic-related air pollution by using modeled source-specific PM_2.5_ concentrations. For PAD we observed even stronger interactions between AAD and traffic-related air pollution when examining gasoline and diesel generated PM_2.5_ as we did when examining residential proximity to roadways. We did also observe an interaction between total PM_2.5_ and AAD in association with PAD (β = 0.09; 95% confidence interval = 0.01 – 0.17; P = 0.02), however after regressing out diesel and gasoline generated PM_2.5_, the remaining PM_2.5_ residuals did not have an interaction with AAD. SBP and DBP were not associated with the PM_2.5_ sources (Supplementary Table 2, Supplementary Table 3). In examining the variance inflation factor (VIF) for signs of multicollinearity we only observed potential multicollinearity (VIF > 5) for interactions with total PM_2.5_. Thus, these primary analyses do not appear to be impacted by multicollinearity. This is further highlighted by the fact that the Pearson correlation (r^2^) between each aging measure (AAD and PhenoAAD) and each exposure considered were all less than 0.0025.

**Figure 2:**
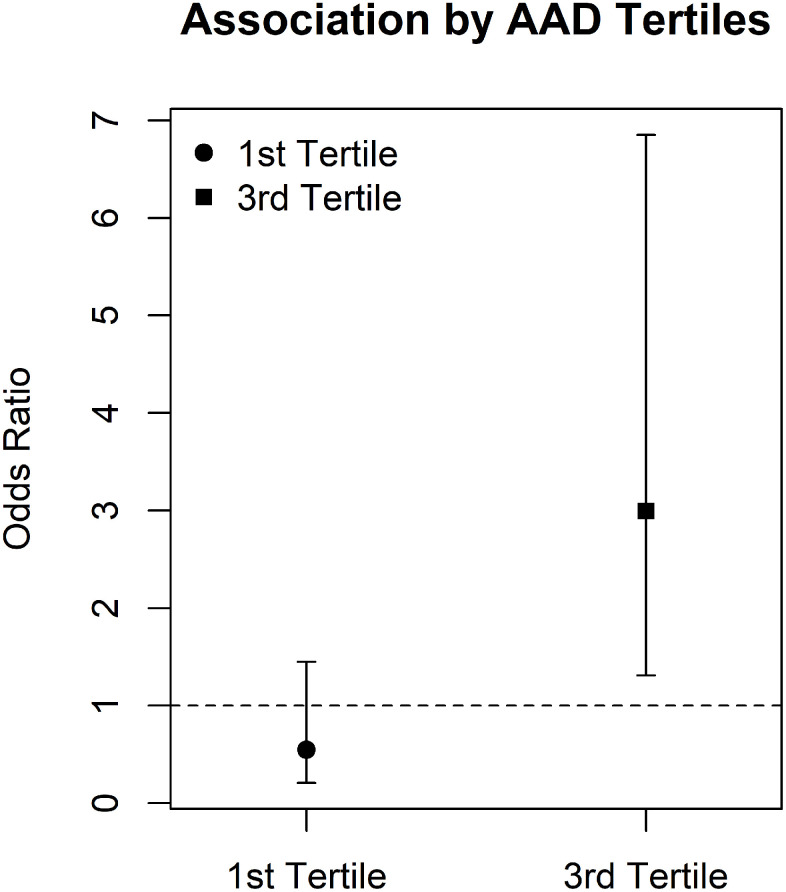
**Association between residential proximity to roadways and PAD by AAD tertiles Interaction between AAD and residential proximity to roadways is visualized by associating residential proximity to roadways in the first tertile of the AAD distribution and comparing association to those seen for the third tertile of the AAD distribution.** AAD = age acceleration difference, PAD = peripheral arterial disease.

As interaction effect estimates can be difficult to visualize, we also examined interactions by classifying individuals based on tertiles of the distribution for each of the accelerated aging parameters and then compared the first (lowest age acceleration) versus third (highest age acceleration) tertiles. We focused these analyses on the AAD-proximity to roadways interaction for PAD as it was the only outcome and interaction that showed an interaction proximity to roadways as well as traffic-related air pollution sources. Associations between PAD and proximity to roadways was effectively null in individuals in the lowest tertile of the AAD distribution (interaction odds ratio = 0.81, 95% confidence interval = 0.34 – 1.90; mean AAD = -2.52 y) while we saw an elevated association in the highest tertile of the AAD distribution (interaction odds ratio = 2.79; 95% confidence interval = 1.09 – 7.11; mean AAD = 11.5 y; Figure 2). The interaction between AAD tertile and proximity to roadways was also significant for PAD (P = 0.03).

## DISCUSSION

Age has long been considered a potential vulnerability factor for environmental exposures, and the elderly can have increased and unique environmental health risks [2]. While chronological age is the standard indicator of age-related risks, recent research has demonstrated that biological age measures have associations with chronic disease and mortality even when controlling for chronological age [8–12]. Our results indicate that accelerated DNA methylation age may be a factor which increases sensitivity to traffic-related air pollution exposure. Chronological age itself did not seem to increase sensitivity in this cohort, further highlighting the potential for biological aging parameters to be more accurate indicators of environmental sensitivity than chronological age in some situations. As PAD is associated with near roadway exposures [13, 17, 18], these interactions may represent a path towards identifying molecular indicators of increased sensitivity to relevant exposures. AAD appeared to be a more robust indicator of environmental sensitivity than PhenoAAD (Table 2). Individuals in the third tertile of the AAD distribution had a significant increased risk of PAD with decreasing distance to roadways, while individuals in the first tertile of the AAD distribution showed no association.

Horvath epigenetic age (the basis for AAD) is designed to estimate epigenetic age in a diverse set of cells and tissues using a single set of 353 epigenetic loci [19], and it has been widely validated in several studies [4]. PhenoAAD is designed to specifically estimate epigenetic age as correlated with several clinical parameters and was developed specifically using blood samples [20]. Both AAD and PhenoAAD are associated with mortality [9, 20]. AAD is associated with multiple clinical outcomes including obesity [10, 21], birth weight [21], hemostasis and blood clotting [12], infection [22], and cancer [8]. PhenoAAD is associated with a smaller number of clinical outcomes [23] likely due to its more recent development. Given the broad association of AAD with several clinical traits, there are a number of biological mechanisms that could explain its interaction with near roadways.

A recent, comprehensive review highlighted the associations between epigenetic age measures and environmental exposures. The chemical, social, and even infectious environment are all associated with epigenetic age and accelerated epigenetic aging [5]. However, this is the first time that a study has highlighted epigenetic age as a potential modifier of the associations between environmental exposures and health outcomes. While genetic variation can modify associations between air pollution and vascular outcomes [24], relatively little has been reported using DNA methylation as a modifier of exposures. Mitochondrial DNA methylation modifies associations between traffic-related air pollution and inflammation biomarkers [25], however, neither AAD nor PhenoAAD are dependent on mitochondrial DNA methylation loci. Also, the genetic variants associated with AAD and PhenoAAD, do not overlap with the genetic variants found to modify associations between near roadway exposures and PAD [26]. Thus, these interactions represent novel interactions inherently linked to a key biological parameter, aging. These associations offer insight into alternative measures of aging, e.g. epigenetic age, which may serve as better biomarkers of environmental sensitivity. After refinement and replication in future studies, these interactions may offer clues into making more personalized environmental health recommendations based on the underlying biology of the individual.

There are several strengths and limitations of this study. At 497 participants, this is a relatively small study. The moderate sample size limits our ability to examine a wider range of outcomes or to examine interactions with extremes of the AAD or PhenoAAD distribution which might be even stronger than the interactions observed here. This study also assumes, that the participants have been long-term residents of their listed primary address. This is a common assumption for studies of long-term exposures, that has been reasonable in similar exposure studies within the CATHGEN cohort [13, 15] and thus we believe to also be reasonable here. While individual-level socioeconomic status was not available in the cohort we did have access to an area-level socioeconomic status indicator that incorporates 11 census variables and has been associated with health outcomes [16]. As a study entirely based within a cardiac catheterization cohort, these results may not generalize to the general population. However, in previous studies associations found in CATHGEN have been similar or stronger than associations found in more general populations [13, 14]. Additionally, underlying cardiovascular disease has also been proposed as a sensitivity factor for environmental exposures, making cardiac catheterization patients a population of interest where air pollution associations may be stronger than the general population.

While proximity to major roadways is a rather coarse indicator of near roadway air pollution exposure, we complemented this with estimates of residential exposure to gasoline and diesel generated PM_2.5_, which may be more direct indicators of traffic-related air pollution exposure. As the resolution of the source-specific PM_2.5_ model was 12km, these measures likely incorporate background air pollution from many nearby sources, as opposed to traffic immediately nearby the residence, as would be captured by proximity to major roads. AAD had interactions with both of these exposures as well as with PM_2.5_, but not with the residuals of PM_2.5_ after regressing out diesel and gasoline sources, suggesting observed interactions may be strongest with traffic-related air pollution if not specific to it. PhenoAAD had interactions with residential proximity to major roadways but not gasoline or diesel source-specific PM_2.5_. This could indicate that PhenoAAD is associated with other aspects of near roadway exposure that are not captured by gasoline or diesel-generated PM_2.5_, e.g. noise. Noise maps are not available for the study area, but noise remains an important exposure worth future exploration. An additional explanation could be that at 12 km the spatial resolution of the diesel and gasoline exposure assessment was not high enough to capture associations with PhenoAAD. Finally, both AAD and PhenoAAD were assessed in blood. While AAD is valid in a wide range of tissues, and typically correlated among tissues from the same individual [19], we are still only able to speak to the discrimination of blood AAD and PhenoAAD for environmental sensitivity, as opposed to other tissues which may be more directly impacted by air pollution exposure. However, blood is often used as a surrogate tissue, particularly for inflammation associated outcomes like PAD and blood pressure, and would be the tissue most likely to be sampled in large studies that seek to understand environmental sensitivity in the broader population.

In all, this study represents an initial examination of the potential for DNA methylation aging biomarkers, to be indicators of sensitivity to environmental exposures. In the case of PAD, there may be a specificity of these interactions for near roadway exposures, however this needs to be validated in large, diverse populations and for a wider variety of outcomes and exposures. The potential for molecular biomarkers to be markers of environmental sensitivity has broad public health and personalized medicine implications including identification of individuals most at risk, targeting of communication and intervention strategies based on individual risk, and narrowing uncertainties in the estimation of the public health impacts of environmental exposures. All of these opportunities should be explored as we seek to understand, inform, and protect the most vulnerable and sensitive populations.

## MATERIALS AND METHODS

### CATHGEN

The Catheterization Genetics (CATHGEN) cohort is a cohort of patients seen at Duke University Medical Center for a cardiac catheterization procedure between 2001 and 2010 [27]. Each CATHGEN participant provided informed consent for the collection of medical data as well as biosamples at the time of catheterization. The study was approved by the Duke University Institutional Review Board. Assessment of DNA methylation was performed on 563 individuals using the Illumina 850k microarray platform using published methods [28]. In previous research, six neighborhood clusters were created in Wake, Durham, and Orange counties, NC in which neighborhoods (census block groups) were clustered based on sociodemographic characteristics [16, 29]. The individuals chosen for DNA methylation assessment were randomly selected from these sociodemographic clusters (~112 per cluster).

### Age acceleration measures

DNA methylation age acceleration is a measure of the difference between age estimated using DNA methylation loci (epigenetic/biological age) and chronological age. It is designed to estimate deviations between biological and chronological age, with positive values indicating age acceleration. We decided to examine two DNA methylation age acceleration measures: age acceleration difference (AAD) and phenotypic age acceleration difference (PhenoAAD). AAD and PhenoAAD are both defined as the difference between their respective epigenetic age estimation measures (DNA methylation age [19] and Phenotypic Age [20] respectively) and chronological age. AAD was developed using the Illumina 450k DNA methylation array platform while PhenoAAD was developed using the Illumina 850k DNA methylation array platform. Both platforms use identical chemistry to determine DNA methylation status, and differ primarily in the number of DNA methylation loci assessed, with the 850k platform assessing nearly twice the number of DNA methylation loci. While there have been reports of underestimation of adult epigenetic age, from which AAD is derived, when assessed using the 850k platform [28] this was revealed to be a shift by a constant in the estimation of this aging parameter which does not bias the performance of this aging parameter in association analyses. In CATHGEN, AAD and PhenoAAD were moderately positively correlated with a Spearman r of 0.60. We elected not to include age acceleration measures derived from the Hannum measure, another commonly used aging biomarker derived from DNA methylation data [30], as they might have DNA methylation platform-specific differences that are dependent on age and thus might bias associations [28].

### Exposures

Our primary exposure was residential proximity to major roadways. We defined major roadways as interstate and state highways and major intra-city arterials in identical fashion to previous CATHGEN publications [13, 15]. Similar to previous publications, we also performed an inverse-log transformation of the exposure as this transformation has been seen to best model near roadway exposure associations in the past [13]. We also examined associations with gasoline and diesel PM_2.5_ sources as sensitivity analyses for the primary analysis. PM_2.5_ sources were assessed using a Chemical Mass Balance [31] model with gas-constrained source apportionment [32]. Data for the model came from the two monitoring networks, the Chemical Speciation Network and the Interagency Monitoring of Protected Visual Environments network, and a chemical transport model, the Community Multiscale Air Quality model (version 4.5) [33]. Use of similar exposure assessment models within the CATHGEN cohort has been previously evaluated and compares well with other PM_2.5_ exposure assessment models [34, 35]. The model generated source apportioned PM_2.5_ estimates for the North Carolina region of the USA from 2002 – 2011. Model estimates of daily and source apportioned PM_2.5_ were provided at a 12x12 km resolution. We computed annual averages for each PM_2.5_ source to assess long-term exposures and required one year of data for annual average estimates. As the resolution of the source-specific PM_2.5_ model was 12km, these measures likely incorporate background air pollution from many nearby sources, as opposed to the model as opposed to traffic immediately nearby the residence, as would be captured by proximity to major roads. Participants were matched to exposure data based on their date of catheterization and primary address at the time of catheterization.

### Analytic approach

We used multiplicative interaction models to determine if there was an interaction between AAD or PhenoAAD and each exposure for three health outcomes: peripheral arterial disease (PAD), systolic blood pressure (SBP), and diastolic blood pressure (DBP). We initially also considered hypertension as an outcome, however logistic regression models involving hypertension were often plagued by near complete separation which could inflate regression coefficients, thus we evaluated SBP and DBP. Outcomes were chosen based on previous associations with traffic-related air pollution exposure in CATHGEN [13]. We examined two models: a full model adjusting for age, race, sex, smoking status, and sociodemographic cluster, and a clinical model which included all the terms of the full model plus diabetes status, body mass index, and history of hyperlipidemia. There were five sociodemographic clusters defined based on Ward's hierarchical clustering of 11 census variables and developed specifically to assess neighborhood socioeconomic status. These have been associated with health outcomes in CATHGEN previously [16, 29] and were the basis for sampling participants, with ~112 participants sampled from each cluster (Supplementary Table 1). Thus, sociodemographic cluster was included as a factor variable to adjust for both socioeconomic status as well as cohort sampling strategy. As a sensitivity analysis to explore the impact of cell proportions we also adjusted analyses for proportions of the following cell types: CD8-T, CD4-T, Natural Killer, B cell, Monocytes, and Granulocytes. AAD has been previously associated with air pollution exposure [5] which means that models could be subject to multicollinearity. We checked the variance inflation factor (VIF) and used a VIF above 5 as an indication of potential multicollinearity.

To better aid in visualizing the interactions, we also evaluated interactions by classifying individuals into tertiles based on their AAD and PhenoAAD distributions. Supplementary Figure 1 shows the distribution of PhenoAAD tertiles for each tertile of AAD. We then evaluated the association between the first and the third tertile of inverse-log transformed distance to major roadways and each outcome (PAD, SBP, and DBP), and calculated an interaction p-value for age acceleration tertile and inverse-log transformed distance to major roadways using a multiplicative model (full model). We did not make this approach the primary approach as it reduces the sample size by a third, by removing the 2^nd^ tertile from analysis, and because continuous traffic exposure metrics have shown better fits than binary measures in previous analyses of the CATHGEN cohort [13].

To examine if interactions were driven primarily by chronological age, we evaluated interactions between residential proximity to roadways and chronological age. We used the full model for confounder adjustment and additionally included adjustment for AAD and PhenoAAD. If our associations are driven primarily by a biological aging phenomenon, as opposed to chronological age, then we would expect attenuated interactions between chronological age and residential proximity to roadways, and little to no association after adjusting for AAD and PhenoAAD.

Prior to analysis all exposures were interquartile range transformed to improve comparability of model effect estimates. Given the single primary exposure and the correlation among epigenetic aging measures and outcomes, we did not impose a multiple testing penalty and instead report on all interactions with P < 0.05 in the full model. As estimates were highly concordant for the full and clinical model, the clinical model was treated as a sensitivity analysis. All models were run in R version 3.5.1 [36]. Logistic regression was used for the binary outcome of PAD, while linear regression was used for the continuous outcomes of SBP and DBP.

### Disclosure

This manuscript does not necessarily represent the views and policies of the US Environmental Protection Agency.

## Supplementary Material

Supplementary Figures

Supplementary Tables
