# Supplementary material for: Accelerated epigenetic age as a biomarker of cardiovascular sensitivity to traffic-related air pollution
Source: Aging (Albany NY). 2020 Dec 7;12(23):24141–55. doi: 10.18632/aging.202341 (PMC7762491; doi:10.18632/aging.202341)
Supplement: Supplementary Figures [file aging-12-202341-s001.pdf]

SUPPLEMENTARY FIGURES

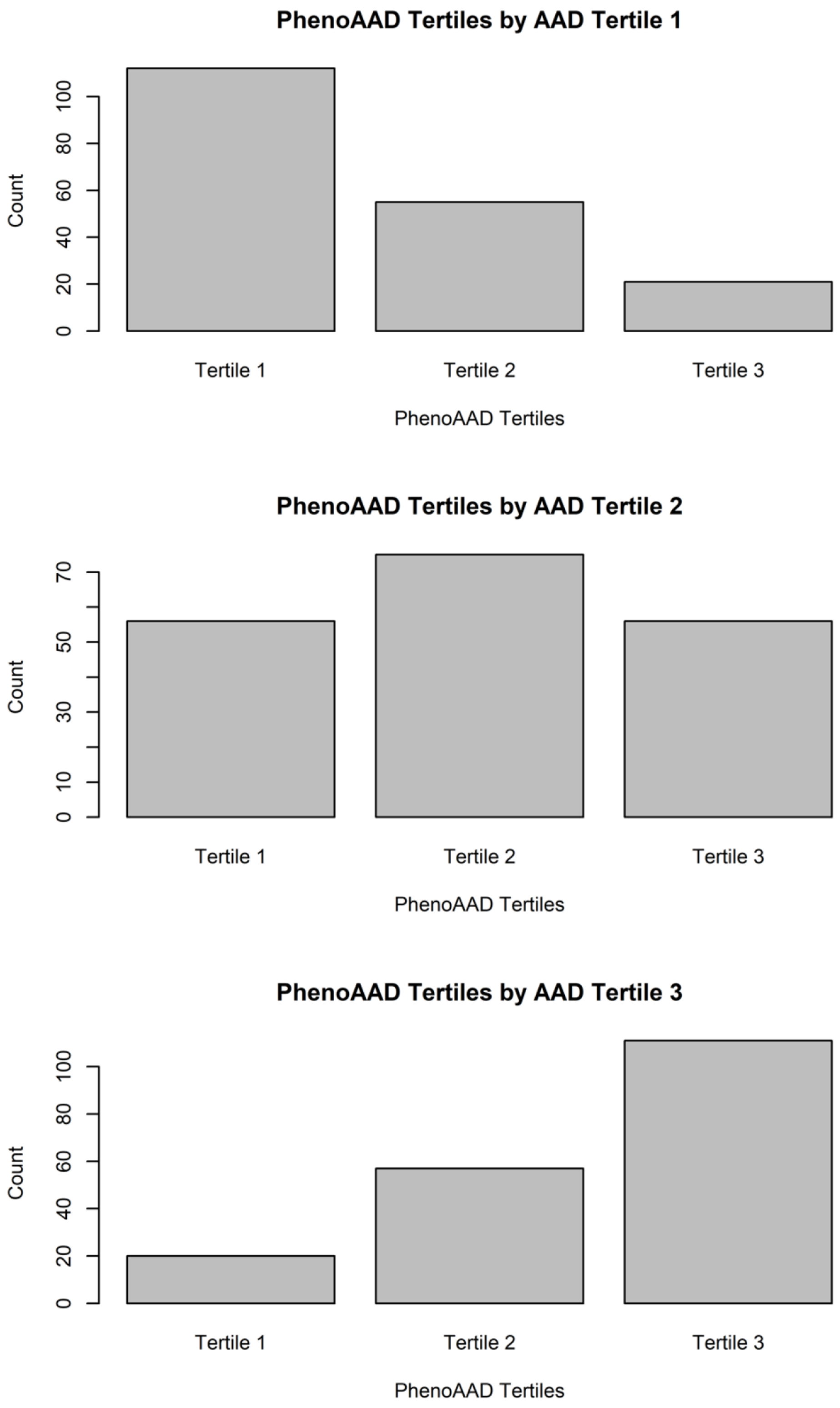

Supplementary Figure 1. Counts of Phenotypic Age Acceleration Difference (PhenoAAD) tertiles by Age Acceleration Difference (AAD) tertiles.

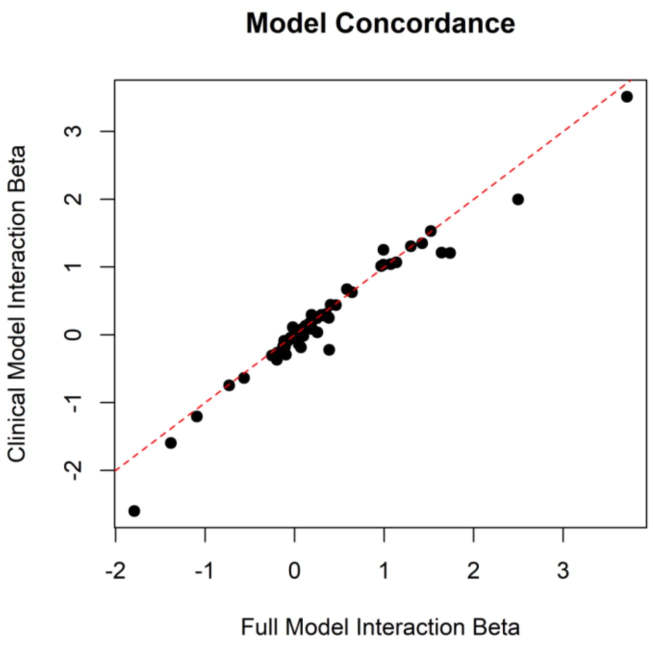

Supplementary Figure 2. Concordance of Models. Model concordance between the Full Model (adjusted for age, race, sex, smoking, and sociodemographic cluster) and the Clinical Model (Full Model + body mass index, history of diabetes, and history of hyperlipidemia) was determined by the correspondence of the exposure-aging interaction term. Plotted above are the interaction term regression coefficients (Beta) for each of the exposures (primary, positive controls, and negative controls) for all outcomes and aging measures. The red line is the y=x line which would indicate identity between the models. The Pearson correlation (r^2) between the models was 0.96.

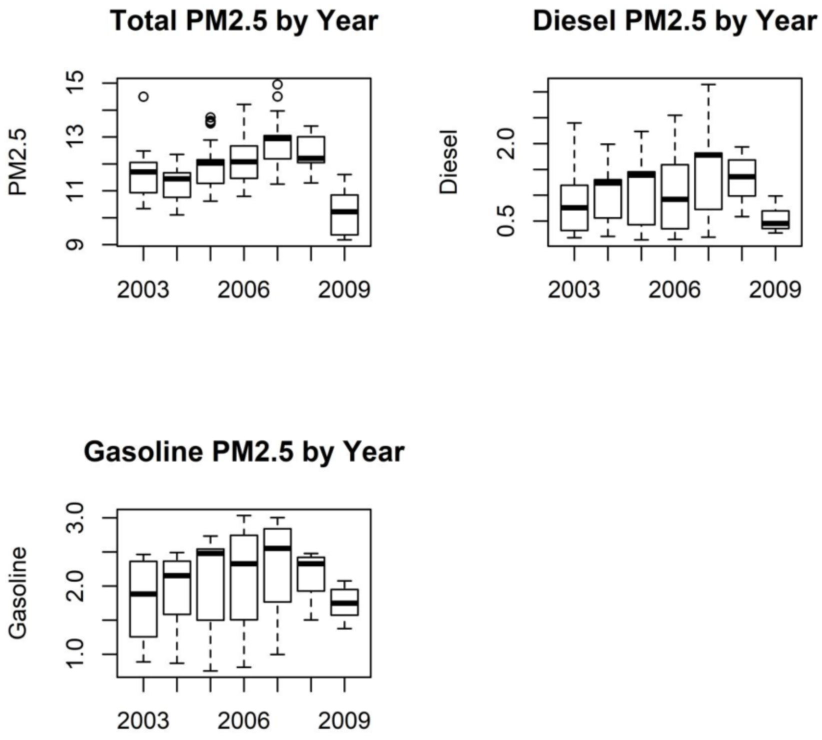

Supplementary Figure 3. Source apportioned $PM_{2.5}$ by year. Boxplots of the source apportioned $PM_{2.5}$ (µg/m^3) by year.
