# Supplementary material for: Accelerated epigenetic age as a biomarker of cardiovascular sensitivity to traffic-related air pollution
Source: Aging (Albany NY). 2020 Dec 7;12(23):24141–55. doi: 10.18632/aging.202341 (PMC7762491; doi:10.18632/aging.202341)
Supplement: Supplementary Tables [file aging-12-202341-s002.pdf]

SUPPLEMENTARY TABLES

Supplementary Table 1. Neighborhood sociodemographic cluster definitions and participant counts.

Sociodemographic cluster	N	Description
Cluster 1	112	Urban with high percentages people who are African-American and are employed in non-managerial positions
Cluster 2	112	Urban, lower income, with a high proportion of residents on public assistance, unemployed and in non-managerial occupations
Cluster 3	112	Less urban than Clusters 1 and 2 with most of the population above the poverty level and unemployed as well as a large percentage of the population having a Bachelor's degree and in non-managerial occupations
Cluster 4	113	Cluster with the highest percentage of residents not identified as African or European American. This Cluster has a high percentage of people with a Bachelor's degree and low unemployment
Cluster 5	114	This Cluster is predominantly rural and has a low percentage of the population living below the poverty line. A low percentage of the population reported being unemployed, most were non-African-American and this cluster has high rates of owner-occupied housing

Supplementary Table 2. Interactions for full and clinical model.

Model	Outcome	Aging	Exposure	Beta	SE	Interaction P
Full	PAD	AAD	Proximity to major roadways	0.06	0.03	0.04
Clinical	PAD	AAD	Proximity to major roadways	0.06	0.03	0.05
Full	PAD	AAD	Gasoline generated PM	0.13	0.05	0.008
Clinical	PAD	AAD	Gasoline generated PM	0.13	0.05	0.01
Full	PAD	AAD	Diesel generated PM	0.13	0.05	0.01
Clinical	PAD	AAD	Diesel generated PM	0.13	0.05	0.02
Full	PAD	AAD	non-traffic PM	0.03	0.04	0.42
Clinical	PAD	AAD	non-traffic PM	0.01	0.04	0.88
Full	PAD	PhenoAAD	Proximity to major roadways	0.04	0.03	0.14
Clinical	PAD	PhenoAAD	Proximity to major roadways	0.04	0.03	0.16
Full	PAD	PhenoAAD	Gasoline generated PM	0.01	0.05	0.84
Clinical	PAD	PhenoAAD	Gasoline generated PM	0.02	0.05	0.74
Full	PAD	PhenoAAD	Diesel generated PM	0.00	0.05	0.98
Clinical	PAD	PhenoAAD	Diesel generated PM	0.01	0.05	0.83
Full	PAD	PhenoAAD	non-traffic PM	-0.01	0.04	0.84
Clinical	PAD	PhenoAAD	non-traffic PM	-0.02	0.04	0.55
Full	SBP	AAD	Proximity to major roadways	0.46	0.20	0.02
Clinical	SBP	AAD	Proximity to major roadways	0.44	0.20	0.03
Full	SBP	AAD	Gasoline generated PM	0.35	0.28	0.21
Clinical	SBP	AAD	Gasoline generated PM	0.31	0.28	0.27
Full	SBP	AAD	Diesel generated PM	0.33	0.28	0.24
Clinical	SBP	AAD	Diesel generated PM	0.28	0.28	0.32
Full	SBP	AAD	non-traffic PM	-0.04	0.21	0.84
Clinical	SBP	AAD	non-traffic PM	-0.04	0.21	0.85
Full	SBP	PhenoAAD	Proximity to major roadways	0.12	0.16	0.46
Clinical	SBP	PhenoAAD	Proximity to major roadways	0.13	0.16	0.41
Full	SBP	PhenoAAD	Gasoline generated PM	0.05	0.24	0.84
Clinical	SBP	PhenoAAD	Gasoline generated PM	0.00	0.23	0.99
Full	SBP	PhenoAAD	Diesel generated PM	0.12	0.23	0.60
Clinical	SBP	PhenoAAD	Diesel generated PM	0.08	0.23	0.73
Full	SBP	PhenoAAD	non-traffic PM	0.08	0.19	0.66
Clinical	SBP	PhenoAAD	non-traffic PM	0.07	0.19	0.71
Full	DBP	AAD	Proximity to major roadways	0.25	0.11	0.03

Clinical	DBP	AAD	Proximity to major roadways	0.24	0.11	0.03
Full	DBP	AAD	Gasoline generated PM	0.17	0.15	0.29
Clinical	DBP	AAD	Gasoline generated PM	0.17	0.16	0.28
Full	DBP	AAD	Diesel generated PM	0.09	0.16	0.57
Clinical	DBP	AAD	Diesel generated PM	0.09	0.16	0.57
Full	DBP	AAD	non-traffic PM	0.06	0.12	0.61
Clinical	DBP	AAD	non-traffic PM	0.07	0.12	0.55
Full	DBP	PhenoAAD	Proximity to major roadways	0.19	0.09	0.04
Clinical	DBP	PhenoAAD	Proximity to major roadways	0.19	0.09	0.04
Full	DBP	PhenoAAD	Gasoline generated PM	0.00	0.13	1.00
Clinical	DBP	PhenoAAD	Gasoline generated PM	0.00	0.13	0.97
Full	DBP	PhenoAAD	Diesel generated PM	0.02	0.13	0.91
Clinical	DBP	PhenoAAD	Diesel generated PM	0.01	0.13	0.95
Full	DBP	PhenoAAD	non-traffic PM	0.09	0.10	0.39
Clinical	DBP	PhenoAAD	non-traffic PM	0.09	0.10	0.39

Interactions for the Full and Clinical model for all exposures, aging measures, and outcomes. Estimates provided are for the interaction between the exposure and the accelerated aging measure given in each row. Non-traffic $PM_{2.5}$ refers to the residuals of $PM_{2.5}$ after regressing out $PM_{2.5}$ from diesel and gasoline sources. AAD = age acceleration difference; Beta = regression coefficient for interaction term; DBP = diastolic blood pressure; PAD = peripheral arterial disease; PM = particulate matter < 2.5 micrometers in diameter ($PM_{2.5}$); PhenoAAD = phenotypic age acceleration difference; SBP = systolic blood pressure.

Supplementary Table 3. Comparison of models with and without adjustment for year of catheterization.

Outcome	Aging	Poll	Full			Full + Year of catheterization		
			Beta	SE	P	Beta	SE	P
DBP	AAD	Diesel generated PM	0.09	0.16	0.57	0.07	0.16	0.66
DBP	AAD	Gasoline generated PM	0.17	0.15	0.29	0.13	0.15	0.39
DBP	AAD	non-traffic PM	0.06	0.12	0.61	0.08	0.12	0.47
DBP	AAD	Total $PM_{2.5}$	0.07	0.12	0.55	0.08	0.12	0.49
PAD	AAD	Diesel generated PM	0.13	0.05	0.01	0.12	0.05	0.02
PAD	AAD	Gasoline generated PM	0.13	0.05	0.01	0.12	0.05	0.01
PAD	AAD	non-traffic PM	0.03	0.04	0.42	0.03	0.04	0.42
PAD	AAD	Total $PM_{2.5}$	0.09	0.04	0.02	0.09	0.04	0.02
SBP	AAD	Diesel generated PM	0.33	0.28	0.24	0.33	0.29	0.25
SBP	AAD	Gasoline generated PM	0.35	0.28	0.21	0.35	0.28	0.21
SBP	AAD	non-traffic PM	-0.04	0.21	0.84	-0.05	0.22	0.83
SBP	AAD	Total $PM_{2.5}$	0.21	0.22	0.33	0.21	0.22	0.33

Year can be an important confounder in some air pollution models as both air quality and clinical factors can vary over years. Here we justify not including year within the models by showing it has little effect on the interaction term in the Full model. As PAD was a binary outcome it estimates are given on the log-odds scale. All estimates are for the aging-air pollution interaction term. Full model adjustment was age, race, sex, smoking status, and sociodemographic cluster. The Clinical model adjustment included all terms for the Full model plus diabetes status, body mass index, and history of hyperlipidemia. Beta = regression coefficient for interaction term; Diesel generated PM = diesel source apportioned PM2.5; Gasoline generated PM = gasoline source apportioned PM2.5; PM2.5 = particulate matter < 2.5 µm in diameter; SE = standard error.

Supplementary Table 4. Full interaction model with additional adjustment for cell counts.

Outcome	Aging	Beta	SE	P
PAD	AAD	0.06	0.03	0.04
DBP	AAD	0.29	0.11	0.01
SBP	AAD	0.55	0.20	0.01
PAD	PhenoAAD	0.05	0.03	0.11
DBP	PhenoAAD	0.19	0.09	0.04
SBP	PhenoAAD	0.14	0.16	0.41

Shown in this table are the interactions between proximity to major roadways (inverse log of distance to major roadway) and the epigenetic aging biomarkers (Aging) for each of the outcomes considered. The model used was the full model with additional adjustment for the following cell types: CD4-T, CD8-T, Natural Killer, B cells, Granulocytes, and Monocytes. AAD = age acceleration difference; Beta = interaction term coefficient; DBP = diastolic blood pressure; P = p-value; PhenoAAD = phenotypic age acceleration difference; SBP = systolic blood pressure; SE = standard error.

Supplementary Table 5. Interactions between chronological age and residential proximity to major roadways.

Model	Outcome	Beta	SE	P
Full + AAD + PhenoAAD	PAD	-0.01	0.01	0.58
Full	PAD	-0.01	0.01	0.62
Full + AAD + PhenoAAD	SBP	-0.06	0.08	0.45
Full	SBP	-0.05	0.08	0.53
Full + AAD + PhenoAAD	DBP	-0.03	0.04	0.44
Full	DBP	-0.03	0.04	0.49

We observed no interactions between chronological age and residential proximity to major roadways (inverse-log transform of distance to major roadways). Each row in the table above represents estimates from the interaction term for the multiplicative interaction between chronological age and inverse-log transform of distance to major roadways for the Model and Outcome listed. Full model adjustment was age, race, sex, smoking status, and sociodemographic cluster. Beta = regression coefficient; DBP = diastolic blood pressure; PAD = peripheral arterial disease; SBP = systolic blood pressure; SE = standard error.
